# Applying Genomic Offsets to Breeding Programmes: Bridging Evolutionary Insights With Practical Applications

**DOI:** 10.1111/eva.70155

**Published:** 2025-10-24

**Authors:** Samantha V. Beck, Samuel A. May, Tony Kess, Ian R. Bradbury, Emmanuel A. Lozada‐Soto, Maren Wellenreuther

**Affiliations:** ^1^ Institute of Freshwater and Biodiversity Conservation, UHI Inverness Inverness Scotland UK; ^2^ Fisheries and Oceans Canada Northwest Atlantic Fisheries Centre St. John's Newfoundland Canada; ^3^ Biology Department Dalhousie University Halifax Nova Scotia Canada; ^4^ USDA‐ARS, National Cold Water Marine Aquaculture Center Orono Maine USA; ^5^ The Center for Aquaculture Technologies San Diego California USA; ^6^ USDA‐ARS, National Animal Germplasm Program Fort Collins Colorado USA; ^7^ The New Zealand Institute for Plant and Food Research Ltd Auckland New Zealand; ^8^ School of Biological Sciences University of Auckland Auckland New Zealand

**Keywords:** adaptation, adaptive breeding framework, climate change, genomic selection, optimum contribution selection, selective breeding

## Abstract

As global temperatures rise and become more variable, the capacity of domestic species to adapt, while maintaining production efficiency, is becoming a pressing concern. In this context, genotype‐by‐environment (GxE) interactions pose a significant challenge for selective breeding, as traits that perform well in one environment may not in another. These interactions complicate the design of breeding programmes that aim to ensure long‐term resilience while optimising short‐term productivity. Genomic Offsets—a metric that can quantify the mismatch between current and future genotype–environment associations, predicting potential genetic mismatch to environmental change—may offer a promising solution. In this perspective piece, we explore potential applications of genomic offsets in agriculture and aquaculture, including their use as tools for risk assessment, selective breeding and cryopreservation. We discuss how genomic offsets can overcome hurdles posed by GxE interactions, addressing practical considerations such as data requirements and methodological frameworks, and needed validation efforts. By predicting genetic mismatches and guiding the selection of individuals best suited for changing environmental conditions, our proposed *Adaptive Breeding Framework* may help breeders proactively enhance the resilience of farmed populations.

## 1 Introduction

The fundamental goal of breeding programmes is the identification and selection of individuals with genetic variation that enhance performance within specific production environments (Goddard and Hayes 2009). By systematically selecting individuals that are predicted to have high genetic merit for desirable traits, these programmes can incrementally and cumulatively improve performance across successive generations adapted to the specific environmental context of the breeding operation (Houston et al. 2020). Increasingly, genomics‐informed breeding practices have dramatically improved the efficacy of trait selection, enhancing growth, survival and disease resistance in domestic populations (Meuwissen et al. 2016). While these genomic‐based breeding programmes have seen great success, they often operate across a wide range of environments, from controlled settings in captivity to natural habitats. Precise control of environments ensures that breeding programmes can maximise performance and achieve consistent results. However, when part of the breeding or growth phase occurs in natural or semi‐natural settings, such as agricultural pastures or marine environments, environmental variation, such as daily, seasonal or interannual changes in climate, can significantly impact production. Furthermore, it is increasingly important to ensure that the genetic selection of breeding populations aligns not only with current environmental conditions but also anticipates future climates (Sae‐Lim et al. 2017). Addressing this challenge is critical to achieving long‐term improvement rates, avoiding bottlenecks caused by short‐term breeding goals that overlook future environmental considerations.

The accelerating pace of climate change has heightened the urgency of breeding for resilient food systems (Afzal et al. 2023). Anthropogenic global change is driving environmental transformations that demand rapid genetic responses over short time frames; a stark contrast to the timescales over which natural adaptations typically evolve. Although phenotypic plasticity—the ability of an organism with a single genotype to change its phenotype in response to different environmental conditions—can facilitate short‐term adjustments to these rapid changes, it is the underlying genetic variation that promotes long‐term resilience (Merilä and Hendry 2014). This is underpinned by both empirical and theoretical work showing that populations can evolve in response to diverse environmental pressures, with genomic approaches advancing our understanding of the genetic basis of climate adaptation (Lotterhos 2023; Peng et al. 2024; Rowan et al. 2021; Scheben et al. 2016). These insights are now starting to be applied to breeding programmes (Laverdière et al. 2022), offering solutions that were previously unavailable for many species. A tested strategy for identifying genotypes and traits adapted to specific climates involves studying variation among populations of the same species that inhabit different environmental conditions (Kawecki and Ebert 2004). This research is predicated on the hypothesis that each population carries adaptations to its local environment. Local adaptation occurs when native genotypes exhibit higher fitness in their home environment compared to non‐native genotypes, often as a result of strong and sustained selection pressures specific to that environment (Kawecki and Ebert 2004). While the existence of local adaptation can be demonstrated using common garden or reciprocal transplant experiments, understanding its molecular basis is particularly useful for species not amenable to laboratory experiments, informing conservation strategies, breeding programmes or predictions about how populations may respond to environmental change (Whitlock 2015). Pinpointing loci that confer local adaptation to climate can reveal critical alleles and traits for breeding climate‐resilient plants and animals (Faye et al. 2019; Olatoye et al. 2018; Tsartsianidou et al. 2021). As such, Genotype‐Environment Associations (GEA) have emerged as a common approach to identify genes linked to fitness in specific environments by correlating allele frequencies with environmental gradients (Coop et al. 2010; Waldvogel et al. 2020).

Genomic Offsets (GO) are a recent offshoot of GEA methods that leverage spatial variation in allele frequencies correlated with environmental variables to quantify the mismatch between a population's current genetic composition and the genotypes predicted under altered environmental conditions (Capblancq et al. 2020; Fitzpatrick and Keller 2015). By integrating genomic and environmental data across time or space, GO estimates the magnitude of allele frequency change required for populations to remain in situ as environments shift, making it a valuable tool for forecasting species' range shifts and identifying populations requiring the most genetic change to adapt to changing climates (Bay et al. 2018; Layton et al. 2021). Although GO is a relatively new concept, initial empirical studies are beginning to explore applications in real‐world contexts (Chen et al. 2022; Rellstab et al. 2021), with experimental studies showing how populations with greater GO estimates often suffer reduced fitness in comparison with those with smaller GO estimates (Capblancq and Forester 2021; Fitzpatrick et al. 2021; Lind et al. 2024). However, the strength of this relationship is often context dependent (Lind and Lotterhos 2025), and some simulations show that larger GO could arise under a positive fitness offset (Lotterhos 2024a). Furthermore, GO models primarily capture patterns associated with local‐foreign relative fitness differences (Lotterhos 2024a), rather than directly estimating home‐away fitness differences. As such, we interpret GO cautiously, avoiding terms like ‘maladaptation’ or ‘vulnerability’ when referring to offset values, because they may not universally indicate reduced fitness outcomes. In recognition of their system specific nature, we discuss the use of offsets to infer relative—not absolute—risks that may inform conservation, management and breeding strategies. Herein, we refer to genomic‐offset models as ‘GO’ and the outputs of these models as ‘GO estimates’ or ‘GO values’. Translating GO insights into selective‐breeding programmes adds another layer of demographic complexity. Although the implementation of GO in breeding contexts remains in its infancy, it nonetheless represents a valuable framework whose development and empirical validation could greatly enhance future genetic‐assisted breeding strategies.

This perspective paper aimed to explore potential applications of GO in plant and animal breeding programmes. First, we outline the general approach to calculating GO, including the necessary assumptions and data requirements for rigorous assessment. Second, we discuss how the concept of GO may be integrated to create an *Adaptive Breeding Framework* to inform breeding programmes to identify and select individuals capable of thriving in changing environments, thereby enhancing the resilience and performance of breeding populations in response to ongoing environmental change. Third, we examine the limitations of this approach and the current challenges that may hinder its integration into breeding programmes. Finally, we propose strategies for overcoming these challenges and advancing the implementation of GO in future breeding efforts.

## 2 Methods for Estimating and Validating Genomic Offsets

GO models rely on integrating both genomic and environmental data to predict genetic mismatches under future conditions. Genomic data typically consists of allele frequency information or genomic variation across loci, while the environmental data often includes variables such as temperature or precipitation. Integrating genomics with environmental variation and projections of future environmental conditions allows GO models to quantify the genetic changes needed for populations to track environmental changes. Depending on the specific conservation or breeding objectives, different types of offsets can be calculated. One such approach involves ‘local offsets’, which provide a space‐for‐time estimate of genomic change required for populations to remain aligned with projected future conditions in their current geographic locations (Fitzpatrick and Keller 2015; Gougherty et al. 2021). Local estimates focus on the degree of instantaneous mismatch between a population's current genetic composition and the environment it will likely face in the future, without accounting for migration or gene flow as processes that could facilitate gradual adaptation. GO can also be adapted to estimate ‘donor’ or ‘recipient’ importance to guide assisted migration efforts (Aitken and Bemmels 2015; Lachmuth et al. 2023). Donor importance identifies seed or propagule sources that are preadapted to future climate conditions, enabling targeted gene flow, while recipient importance is analogous to future habitat suitability, with the environment that yields the lowest GO value representing the most favourable location for relocating the population of interest (Lachmuth et al. 2023).

GO can provide a more efficient alternative to traditional genetic assessment methods such as common garden experiments or provenance trials, which can be costly, labour‐intensive, difficult to replicate and may be influenced by short‐term plasticity rather than genetic adaptation. However, traditional genetic assessments remain essential and should be used in parallel with GO estimates to provide a more comprehensive understanding of population status and resilience, with both approaches serving as complementary tools. When implementing GO models, it is common to focus on putatively adaptive loci; yet, empirical and simulation studies suggest that the use of a random set of loci can yield a similar model performance compared to those identified by association methods (Lachmuth et al. 2023; Lind and Lotterhos 2024). Nevertheless, the use of putatively adaptive loci avoids potential confounding effects from nonadaptive loci. Genome‐wide associations (GWA) can be used to identify loci associated with environmentally linked traits (such as growth, disease resistance, phenology, thermal tolerance) for use in GO estimates, offering more informed predictions of a population's genetic capacity to adapt to environmental change based on specific genetic architectures. For traits that are highly plastic (i.e., exhibit significant genotype‐by‐environment (GxE) interactions), identifying loci associated with environmental gradients (GEAs) may be more effective (Capblancq et al. 2020).

The choice of environmental variables can have a significant impact on GO estimates, so inclusion and appropriate scaling of environmental variables should be carefully considered. Identifying which variables are most important in shaping genetic variation across the landscape remains challenging, particularly when variables are highly correlated or when limited associations with the environment exist. Although PCA is a common method for reducing environmental variation into uncorrelated axes, it often lacks ecological interpretability and may overlook nonlinear relationships between environmental variables and genetic variation. More advanced techniques, such as Gradient Forest (GF; Ellis et al. 2012), offer a powerful approach for identifying environmental variables that explain the greatest proportion of genetic variation. As an ensemble machine‐learning method, GF handles correlated variables by permuting out‐of‐bag samples within partitions of correlated predictors (Strobl et al. 2008), enabling the assessment of the overall importance of predictors despite their correlations. This targeted selection of variables contributes to more accurate GO estimates and provides a clearer understanding of the environmental pressures driving genetic adaptation in natural populations (Lind and Lotterhos 2024). Notably, these methods have yet to be tested in domestic lines, and it remains uncertain whether specific domestic populations exhibit sufficient genetic variation and environmental gradients to make these approaches applicable without the use of additional data from natural populations.

The emergence of large‐scale genomic datasets in wild populations across species of domestication interest has the potential to aid in detecting climate and ecologically relevant loci for monitoring in breeding programmes. Together with broadly available climatic datasets, the first goal of applying GEA methods to breeding programmes is the detection of loci that are relevant to climate adaptation and have been shaped by selection across time. Two methodological considerations need to be made in these analyses: the selection of environmental variables and the selection of the GEA approach. Using environmental variables that are likely to reflect the variability of environmental conditions experienced by domesticated populations can aid in the targeted detection of variation relevant for production goals. Wild populations are likely the best case for testing for the presence of climate‐associated alleles, as they have been shaped by long‐term environmental gradients. In contrast, such gradients are likely to be disrupted in established domesticated lines, which have been subjected to more recent and intensive selection pressures. However, for some domestic species, wild populations do not exist or do not occur along sufficient environmental gradients to resolve genomic environmental relationships. The ability to conduct common garden experiments across divergent domestic lines and resolve the genomic basis of differential performance may provide an alternative and perhaps complementary method. GEA method selection can also impact the set of detected loci; for example, population structure correction can have a significant impact on the detection of adaptive loci (Lotterhos 2023), though not necessarily on the predictive accuracy of these loci (Lachmuth et al. 2023). Following the detection of climate‐relevant loci, different methods can be used to predict how this variation will shape future climate responses in domesticated populations.

Several distinct methodological approaches have been developed to calculate GO (Table 1). While a comprehensive review of each method is beyond the scope of this perspective paper, we provide a brief summary of their key principles and applications. First is the risk of nonadaptiveness (RONA; Rellstab et al. 2016), a univariate method that uses linear regressions for each SNP and environmental factor. Redundancy analysis (RDA; Capblancq and Forester 2021) is another linear method, employing a multivariate ordination framework to model linear relationships among environmental predictors and genomic variation, identifying co‐varying allele frequencies associated with the multivariate environment. The generalised dissimilarity model (GDM; Ferrier et al. 2007) and GF (Ellis et al. 2012; Fitzpatrick and Keller 2015) are nonparametric regression‐based approaches that model compositional turnover of allele frequencies using nonlinear functions of environmental gradients. GDM uses distance matrices to model compositional turnover (‘dissimilarity’), whereas GF employs machine‐learning with ensemble decision trees. Finally, a more recent nonparametric and multivariate geometric GO has been designed that calculates the quadratic distance between vectors of environmental predictors (current and future) based on the effect sizes of specific allele frequencies (Gain et al. 2023).

**TABLE 1:** Summary of methods for calculating genomic offsets (GO), including whether they are parametric, the type of analysis (univariate or multivariate), analysis approach, ability to correct for population structure, as well as key strengths, limitations and references.

Method	Parametric	Univariate/multivariate	Analysis type	Population structure correction	Strengths	Limitations	References
**RONA**	Yes	Univariate	Regression‐based	No	Simple and interpretable results; Effective for small datasets	May not capture complex relationships; Sensitive to outliers	Rellstab et al. 2016
**RDA**	Yes	Multivariate	(Constrained) ordination	Yes—via inclusion of conditioning variables (e.g., PC axes describing population structure)	Handles multiple response variables; Accounts for population structure	Assumes linear relationships; Sensitive to collinearity	Capblancq and Forester 2021
**GDM**	No	Multivariate (compositional turnover)	Distance‐based (FST) dissimilarity	No	Captures complex relationships; Flexible with different data types	Limited interpretability of dissimilarity metrics	Ferrier et al. 2007
**GF**	No	Multivariate (compositional turnover)	Machine‐learning	No—although indirect inclusion of PCs describing population structure could be incorporated as additional environmental variables	Models complex interactions	Requires large datasets; Lacks direct biological interpretability due to unitless distances	Ellis et al. 2012; Fitzpatrick and Keller 2015
**Geometric GO**	Yes	Multivariate	Quadratic distance	Yes—via incorporation of latent factors	Robust to unknown causes of adaptation	Assumes unknown causal variables can be inferred	Gain et al. 2023

Abbreviations: GDM, generalised dissimilarity modelling; GF, gradient forest; PC, principal component; RDA, redundancy analysis; RONA, risk of nonadaptiveness.

**FIGURE 1:**
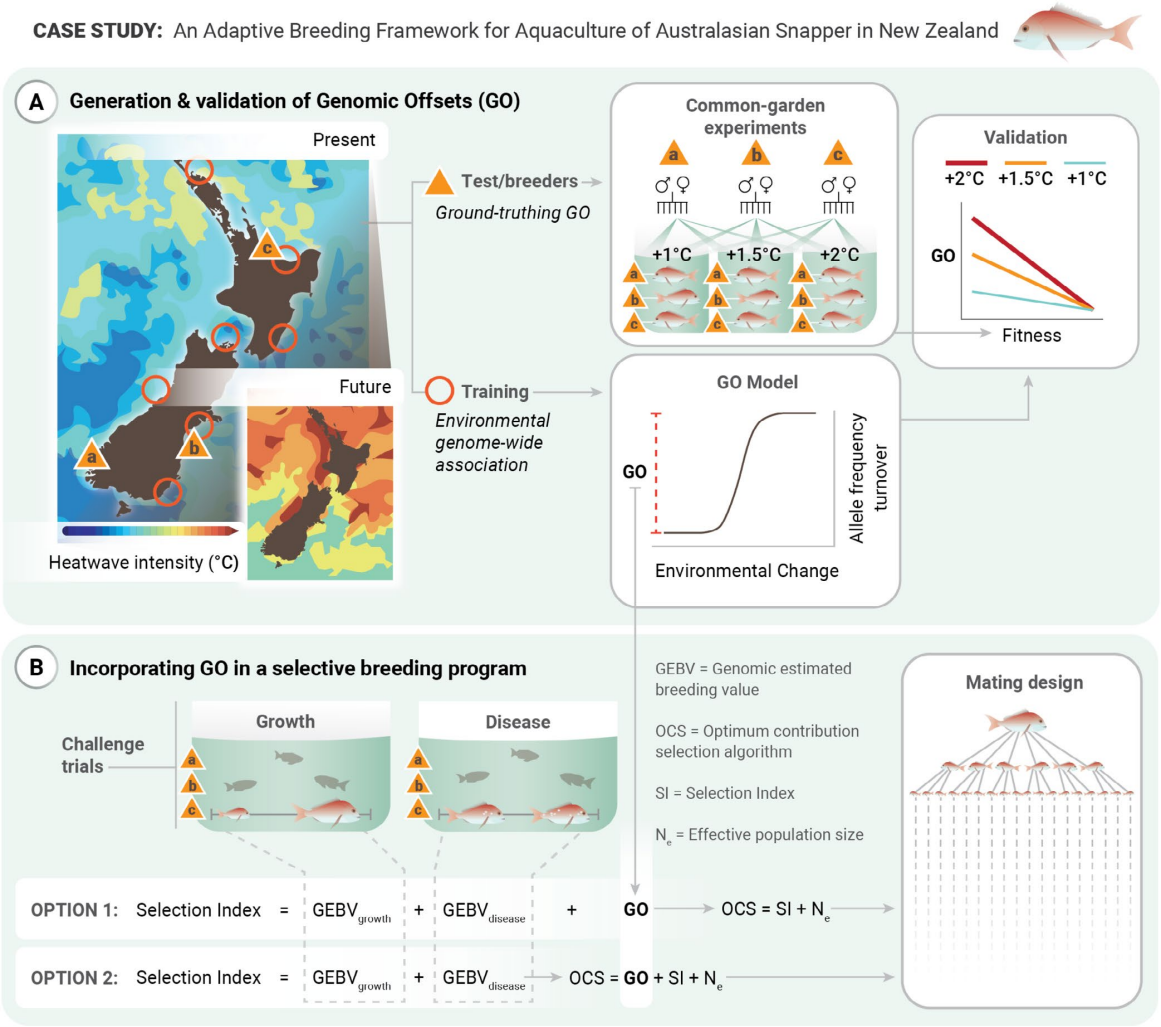
Example of an *Adaptive Breeding Framework* for the Australasian snapper ( *Chrysophrys auratus* ) including: (A) the quantification of genomic offsets from training and test populations in present and predicted future environments and the evaluation of these estimates using common garden experiments; (B) two options for using genomic offsets to inform mating designs in a genomics‐informed selective‐breeding programme.

Although the use of GO to evaluate the extent of genetic change is increasing, evaluations of these estimates remain rare. A key assumption of GO is that populations are optimally adapted to their current environments, which may not always be the case (e.g., Browne et al. 2019). Furthermore, while it is assumed that high GO estimates suggest population vulnerability to environmental change, it has been shown in simulations that, under some conditions, high offsets can also arise from positive fitness changes (Lotterhos 2024a), highlighting the importance of evaluation through experimental data (Lotterhos 2024b). For example, previously collected data from provenance trials, challenge experiments and genetic data can be invaluable to ground‐truth predictions in real‐world scenarios by correlating GO estimates with fitness trends. Validation in plants and invertebrates often employs common garden experiments in multiple environments, providing a controlled method to directly observe how different genotypes perform under various environmental conditions (Capblancq et al. 2023; Chen et al. 2021; Morales‐Cruz et al. 2023) and to identify the best genotypes for specific localities/conditions. However, validation efforts in vertebrates are particularly scarce, often relying upon historical rather than experimental data to correlate estimates with demographic patterns, such as population size (Bay et al. 2018; Miller et al. 2024; Zhou et al. 2024). An important caveat of using population size as a tool to validate GO estimates is the consideration of the effects of small population size, where changes in allele frequencies are likely to reflect patterns of genetic drift, making predictions derived from allele frequency turnover less reliable (Láruson et al. 2022). When designing validation experiments (Lotterhos 2024b; Figure 1a), it is important to tailor GO models to specific needs, such as whether the focus is on conservation efforts aimed at assessing genetic mismatches to future environmental changes or on agricultural applications that seek to identify optimal seed sources for growth and yield in specific environmental conditions.

GO have been applied in various contexts (Bay et al. 2018; Camus et al. 2024; Chen et al. 2022; Hung et al. 2023; Jia et al. 2019; Lachmuth et al. 2023; Peng et al. 2023; Varas‐Myrik et al. 2024; Zhang et al. 2023), each with distinct goals for their predictions. For instance, in conservation, the aim is often to assess how maladapted a population is to future environmental change and, based on that, to inform management actions (e.g., Tigano et al. 2024), including assisted migration (i.e., red spruce ( *Picea rubens* ): Lachmuth et al. 2023), assisted gene flow (i.e., South American conifer ( *Araucaria Araucana* ): Varas‐Myrik et al. 2024), identification of donors for evolutionary rescue (i.e., Green‐backed tit ( *Parus monticolus* ) and Elliot's laughingthrush ( *Trochalopteron elliotii* ): Chen et al. 2022), selection of preadapted seed sources (i.e., Chinese arborvitae ( *Platycladus orientalis* ): Jia et al. 2019), protecting habitats (i.e., Rosewoods ( *Dalbergia cochinchinensis* and *Dalbergia oliveri*): Hung et al. 2023), pathogen resistance (i.e., grapevine ( *Vitis arizonica* ): Morales‐Cruz et al. 2023), invasion risk assessment (i.e., fruit flies ( *Bactrocera tryoni* ): Camus et al. 2024; ascidian ( *Molgula manhattensis* ): Chen et al. 2024) and planning restoration programmes (i.e., red spruce ( *Picea rubens* ): Lachmuth et al. 2023). An alternative approach that is applied in forestry is the ‘Climate Based Seed Transfer’ (CBST) system, which incorporates climate change projections into seed sourcing (O'Neill et al. 2017). Traditionally, seed transfer relied on sourcing seeds from local populations, but this method is no longer effective, as local seeds often fail to match future climate conditions, leading to reduced productivity. The CBST system was developed to address this issue, using climate projections to select seed sources that are better adapted to future environmental conditions, rather than relying solely on local origins. Unlike CBST, the application of GO offers a more forward‐thinking approach by quantifying the genetic differences between current and predicted future environments, enabling managers to identify populations requiring the most genetic changes and prioritise those with genetic potential for future resilience (i.e., least amount of genetic change). In agriculture, the goal is typically to determine the most suitable seed sources (i.e., genotypes) for optimal resilience in a particular environmental setting. To date, the use of GO in agriculture has provided valuable insights into the impact of environmental change on crop cultivation and helped to inform assisted migration. For example, Rhoné et al. (2020) found that varieties of pearl millet with the highest GO (i.e., highest amount of needed genetic change) also exhibited the largest yield loss, highlighting the need for managers to source seeds outside their current geographical range to ensure that collected germplasm is suitable for cultivation in the future. While GO models have been applied at the population level, using GO models to generate individual‐level predictions would be a promising, yet undeveloped, research direction. Advancing this approach could significantly enhance our ability to predict and manage individual responses to environmental changes, as well as providing a powerful tool for breeding purposes.

## 3 Applying Genomic Offsets in Selective‐Breeding Programmes

### 3.1 Overview of Challenges and Current Methods

Incorporating GO more directly into breeding programmes could improve the production of farmed species by identifying preadapted genotypes that are well suited to performance in contemporary or future climates. This approach may improve the viability and resilience of breeding stocks, ultimately producing more robust and adaptable agricultural varieties (Varas‐Myrik et al. 2024). As highlighted, GxE interactions represent a prominent logistical hurdle for agricultural advancement. Additionally, with the increasing availability of genomic selection methods (Meuwissen et al. 2016), selective‐breeding programmes are rapidly expanding in terms of the number of traits included and their complexity (Goddard and Hayes 2009). Modern multitrait genomic selection indices, for instance, often comprise a range of quantitative traits such as growth, disease resistance and product quality attributes (i.e., colour, texture and flavour). However, the push for enhanced selection efficiency and intensity may come at the cost of adaptively important genetic variation. As climate change advances, there is an increased risk for latent deleterious effects to emerge, potentially jeopardising animal well‐being and future food security (Benitez‐Alfonso et al. 2023). For example, a breeding programme selecting for improved growth may inadvertently remove important genetic variation associated with disease resistance, especially if the disease is negatively correlated with growth or is only a concern in warmer climates (Piazzon et al. 2020). Therefore, it is critical that breeding programmes consider genomic approaches that not only improve economic traits but also maintain or enhance the adaptive capacity of breeding populations by adopting proactive breeding strategies to address future environmental changes (Razzaq et al. 2021).

Application of environmental genomics to breeding programmes is significantly impeded by lack of knowledge around how genomic variation in domestic lines relates to the environments under which they originally evolved. Both GO and GEA methods rely on the presence of local adaptation. However, in crops and livestock, it is often unclear whether such local adaptation exists, particularly in long‐domesticated lines where strong artificial selection and genetic drift may have decoupled genotypes from the original environmental context. Moreover, these methods can be confounded by population structure or stochastic processes, which can produce spurious correlations between genetic and environmental variables that may not be causally linked to adaptation (Sul et al. 2018). Detecting true signals of environment‐driven selection thus requires not only robust sampling designs and/or statistical controls that account for confounding factors like population structure and drift, but also biologically informed interpretation (Rellstab et al. 2015). Yet the environmental conditions from which domestic populations are drawn are often unknown or come from an unrepresentative subset of the total environmental and ecological variation experienced in the wild. Laboratory environments are often a poor proxy for these environments, due to the missing biotic and simplified abiotic gradients, limiting the utility of these experiments to genomically predict performance in more complex environments. Additionally, long‐established domestic lines may lack genetic variation relative to wild populations due to the combined action of domestication selection and strong genetic drift following genetic bottlenecks, restricting the genetic substrate from which to identify genomic associations. In many breeding contexts, there may have been limited opportunity for local adaptation to arise in the first place. For example, selection may primarily focus on yield or aesthetic traits rather than environmental resilience, and agronomic practices such as irrigation or fertilisation may have buffered environmental stressors that would otherwise drive adaptation. As a result, some domesticated populations may show little or no genetic signal of adaptation to local climatic conditions, limiting the power of environmental association methods. While modern agro‐industrial breeding promotes genetically uniform cultivars, traditional domesticated crop varieties have evolved in diverse environments over millennia, retaining a broad genetic base and promoting resilience to a range of environmental stressors (Bellon et al. 2018; Brush 1995; Li et al. 2024; Salgotra and Chauhan 2023). These comparative levels of diversity between modern and traditional cultivation methods highlight the importance of considering the breeding history of populations when applying GO, as the presence and strength of local adaptation may vary widely across breeding systems. Together, these limitations may strongly bias detected environmental associations in domestic lines, further highlighting the importance of experimental validation of GO and GEA applications (Rêgo et al. 2025; Wu et al. 2025). Broadly, we anticipate our suggested approaches will be most directly applicable to more recently domesticated species (i.e., aquaculture species) or those with populations distributed across wide environmental gradients. Nevertheless, they can also inform strategies involving the use of wild genetic material to enhance resilience in domesticated populations, particularly when environmental variation within domesticated lines is limited.

In this perspective, we discuss several methods by which GEA in natural populations can be leveraged to inform captive breeding programmes, but first we must acknowledge the various ongoing efforts already being used to address emerging climate challenges. One example of a long‐standing strategy to cope with environmental variation is the breeding of multiple strains adapted to different conditions. However, operating selective‐breeding programmes is both costly and time‐consuming, so while this strategy may be suitable for global and large‐scale breeding programmes like cattle, it is inefficient for smaller or emerging markets. Another approach is to use GEA‐type studies to quantify and breed for phenotypic plasticity, tolerance, or resilience as independent, quantitative traits, such that a single strain may perform well in diverse environments (Cowling et al. 2019; Rockett et al. 2023). For example, in dairy cattle breeding, recent efforts have resulted in the development of resilience indicator traits that measure the ability of cows to limit disturbances in production as a result of environmental or disease challenges (Maskal et al. 2024; Poppe et al. 2022). Look‐ahead selection, an optimisation algorithm to enhance long‐term performance of crosses in a prespecified future generation by assessing trade‐offs of breeding for multiple traits (which can include climate tolerance), is another method that bears similarities with GO methods but does not explicitly consider impacts from environmental change (Moeinizade et al. 2019; Zhang and Wang 2022). Finally, cryopreservation technologies are rapidly being adopted, a strategy which allows breeders to safeguard genetic variation that may prove useful in future conditions or with future changes to breeding goals (Blackburn 2012; Blackburn et al. 2024; Long et al. 2019; Wylie et al. 2023).

Several more traditional population genetic approaches should also be considered in tandem with the GO methods we discuss in detail here, particularly when leveraging adaptive diversity from natural populations. For example, Schmidt and Russello (2025) showed in American pikas ( *Ochotona princeps* ) that northern populations, where climate is warming almost four times faster than the global average (Rantanen et al. 2022), exhibited high genomic‐offset values but also harboured greater adaptive diversity, whereas high‐altitude populations displayed the opposite pattern: low offset but high diversity. This contrast demonstrates that the use of GO on its own might be misleading without careful ecological and demographic context. As genetic diversity is predictive of adaptive capacity (Kardos et al. 2021), one simple approach to harness wild adaptive variation for domesticated lines is to compare diversity at climate‐associated loci. However, peaks of association can often span large genomic regions capturing not only causal variants but also linked neutral sites (Schaid et al. 2018), potentially obscuring the true relationship between allele frequencies and environmental adaptation. Complementary approaches, such as common garden experiments in multiple environments or functional validation methods (e.g., gene expression or genome editing), can help validate whether putative adaptive loci truly influence phenotypic traits or fitness across environments, and thereby refine inferences about local adaptation and adaptive capacity. Metrics such as nucleotide diversity, runs of homozygosity and observed heterozygosity can all provide clues into the extent that domesticated populations can capture necessary adaptive diversity from the wild. Greater diversity at climate‐relevant loci may suggest greater potential for adaptation (Uchiyama et al. 2025). Comparative methods in population genomics, such as differentiation statistics (e.g., F_ST_) and ordination methods (e.g., PCA, DAPC), may also be used to identify natural populations and environments that differ the least from the genetic composition of the current domesticated line. These comparative methods can help pinpoint the most genetically similar wild populations, which are likely to represent the original source or closely related gene pools. Such populations may serve as important reservoirs of adaptive variation, especially for traits such as climate resilience, and could be used to guide cryopreservation or introgression efforts aimed at improving the adaptive potential of domesticated stocks without introducing large genomic mismatches (Tymchuk et al. 2007). Alternatively, more divergent wild populations may be targeted when specific adaptive traits, such as heat tolerance, might be required to prepare domesticated lines for future environmental conditions (Molero et al. 2023). Last, genomic prediction approaches using machine learning and polygenic scores can also be used to predict the environmental variation domesticated lines are likely to match based on genotype. For example, in wild populations of Atlantic salmon, polygenic prediction of population decline risk has been conducted and validated using both historical and contemporary samples for estimates of population declines using genomic data (Lehnert et al. 2019). However, these predictions have yet to be validated with demographic data. Leveraging other features of wild population genomic data sets may also prove valuable in risk‐proofing domesticated lines. With these existing strategies in mind, we propose several potential use cases for GO to advance selective‐breeding programmes.

### 3.2 GO as a Risk Assessment Metric

Population‐level GO (as opposed to individual‐level offsets, discussed below) offers a novel metric for assessing the adaptability of breeding lines across varying environmental conditions. By evaluating the genomic potential of populations to cope with future climate scenarios, breeders may better predict which lines will maintain productivity as conditions change. At present, the genetic diversity of a breeding population (e.g., as measured by its effective population size, *N*_e_) is often used as an approximation metric for adaptive capacity. However, GO could offer a more targeted ‘litmus test’ for detecting potential genomic mismatches under specific anticipated future conditions. Quantifying GO values annually, alongside metrics like inbreeding rates and diversity estimates (e.g., observed heterozygosity, nucleotide diversity and effective population sizes) enables the tracking of genomic shifts relative to changing climate projections. While the rate of genetic change may be slow, climate change projections are continuously improving, and strong selective breeding can drive rapid and sometimes unanticipated genomic changes. Therefore, periodic evaluation of GO values helps assess genetic shifts over time and allows for the evaluation of past breeding successes, ensuring that breeding programmes remain responsive to emerging environmental conditions.

In addition, GO can assist growers in making more informed decisions about the best sites and times to raise their existing lines, considering local environmental variation. For example, a shellfish grower evaluating several potential coastal sites to invest in large‐scale aquaculture infrastructure can use environmental factors such as temperature, salinity and pH to quantify GO estimates. By selecting the site where the GO values of their strains are lowest, the grower can optimise survival and performance in that particular environment. This scenario also applies to future‐proofing breeding lines, selecting those environments where GO values are lowest in the next 20–40 years' time. However, caution is warranted when extrapolating into the distant future, especially with range‐edge populations, topographically uniform landscapes and/or using worst‐case socioeconomic scenarios, since the emergence of novel climates presents a serious challenge for accurate projections of ecological responses (Lachmuth et al. 2023; Lind and Lotterhos 2025; Mahony et al. 2017). Combining such GO estimates alongside *N*_e_ and insights into where future conditions represent novel environments can help refine projections, offering a more nuanced understanding of potential risks and more effectively informing breeding and conservation strategies.

### 3.3 GO to Inform Selective‐Breeding Decisions

Building on the previous example, this section outlines how GO can be applied to inform selective‐breeding decisions through the application of an *Adaptive Breeding Framework*, as outlined in Figure 1. After identifying potential alternative sites and their corresponding environmental conditions, managers can estimate GO values and validate these predictions through challenge experiments or provenance trials (Figure 1a). Once validated, there are two main ways GO can be incorporated into the *Adaptive Breeding Framework* to inform selective‐breeding decision‐making.

The first method involves treating individual‐level GO estimates as an independent trait within a standard multitrait genomic selection index (Option 1, Figure 1B). Although methods for estimating individual‐level GO values remain undeveloped, particularly in terms of quantifying uncertainty and bias, this approach offers a promising framework for integrating long‐term resilience into breeding programmes. By standardising or transforming GO values to a similar scale as Genomic Estimated Breeding Values (GEBVs) and adjusting index weights to accommodate different breeding priorities, this method could be a relatively simple addition to current breeding programme workflows. By doing so, breeding programmes could produce selection indices that balance short‐term productivity gains (i.e., growth or disease resistance) with long‐term genomic alignment to future conditions, helping to facilitate resilience. For instance, individuals with low GO values might be prioritised in breeding decisions, ensuring that selected traits are suited for anticipated future environmental scenarios. GO could also be treated as several independent traits; for example, to incorporate several GO estimates from different sets of anticipated future environmental conditions. This approach may help manage the trade‐offs between immediate genetic gains and long‐term genomic resilience, fostering more robust breeding strategies. The selection index resulting from the combination of GEBVs and GO may then be treated as any other selection index, for example, to parameterise Optimum Contribution Selection algorithms (OCS) and inform mating designs. Much work has been done to improve the accuracy of genomic predictions through novel algorithms or enhanced data quality controls; future investigations should similarly focus on establishing robust approaches for estimating individual GO values and for quantifying and integrating the uncertainties associated with both GEBVs and GO.

An alternative method involves incorporating GO directly into OCS algorithms (Option 2, Figure 1B). OCS algorithms look to optimise the genetic contributions from a set of breeders to achieve a desired outcome in one or more population parameters (Clark et al. 2013). While OCS has been traditionally used to maximise genetic gain while constraining levels of kinship or inbreeding, it can also be used to manage genomic inbreeding at a genome‐wide or region‐specific level, maintain levels of effective population size and recover lost genetic ancestry of endangered populations (Toro et al. 2014; Wang et al. 2017). We propose that incorporating GO into these algorithms could prove valuable in optimising strains for anticipated future conditions. Either individual‐ or population‐level GO estimates could be incorporated into OCS: Individual‐level GO values might be treated similarly to how GEBVs are used in contemporary OCS, where individuals with higher GEBVs (lower GO values) are prioritised for breeding. Alternatively, population‐level GO metrics may be iteratively recalculated from genomic data, akin to how effective population sizes are currently used in OCS to optimise for population‐level genetic variation (Wellmann 2019). Successful integration of GO into OCS algorithms would allow breeders to optimise the selection process by ensuring selected crosses not only produce desirable traits in the present (through GEBV optimisation) but also preserve alleles critical for future environmental conditions (by GO optimisation). Currently, no OCS programmes incorporate GO, and we advocate for the development of such integrations to advance and future‐proof breeding strategies. Notably, either individual‐ or population‐level GO may also be used to select wild individuals or populations to incorporate into captive lines, which can be viewed as a form of assisted migration or genetic rescue to increase beneficial genetic variation in lines identified as at‐risk of adverse climate impacts. Assessing the benefits and potential drawbacks of each option should be a future research priority and would perhaps be best accomplished with simulation‐style approaches.

### 3.4 GO for Cryopreservation and Conservation

Cryopreservation of eggs, sperm or embryos is becoming more accessible in many species and presents a useful tool for preserving genetic diversity. Cryopreserved gametes offer a failsafe or insurance strategy, allowing breeding programmes to re‐access lost genetic diversity in the breeding strain, or return to an earlier state if environmental conditions or breeding goals change. However, cryopreserving every individual in a breeding programme is rarely cost‐effective, particularly in large‐scale breeding operations. GO may provide a strategic framework for deciding which individuals or populations should be cryopreserved to ensure future genetic resilience. Unlike the direct integration of GO into breeding programmes, cryopreservation has the benefit of maintaining a reservoir of genetic diversity that can support future environmental conditions, without sacrificing short‐term genetic gains.

Furthermore, GO can guide the selection of wild populations for cryopreservation and conservation breeding programmes. In the context of ongoing climate change, it is crucial to determine which populations to conserve or use in evolutionary rescue or assisted gene flow efforts (e.g., Lachmuth et al. 2023; Varas‐Myrik et al. 2024). By identifying populations with low GO values, conservationists can prioritise those that possess genetic variation essential for adjusting to future environmental conditions. In addition, GO can also be used to identify populations potentially at risk of genetic mismatch with the environment and target them for conservation or cryopreservation in case reconstitution of the population is needed. This targeted approach ensures that valuable genetic resources are strategically preserved and can be utilised in assisted migration or evolutionary rescue initiatives to enhance threatened populations or recolonise extirpated populations and newly available habitats.

## 4 Limitations, Challenges, and Future Directions

Despite the potential benefits of integrating GO into selective‐breeding programmes, several limitations and challenges should be considered that may slow their widespread adoption. GO methodologies are still in their infancy and are only recently transitioning from theoretical and simulation‐based studies to empirical applications. However, empirical studies using GO are presently limited mainly to conservation applications, and no infrastructure yet exists to tailor GO models to breeding programme workflows on an individual scale. Additionally, the need for empirical evaluation of GO estimates is integral to ensure that GO‐informed breeding decisions perform as predicted, and if not, to understand what is causing any deviations. Such evaluations may involve long‐term, longitudinal studies which can be time and resource intensive. However, many industries are likely to have long‐term genetic and environmental data from previous challenge experiments that could be leveraged to validate contemporary GO estimates. These types of evaluations are already routine in many breeding programmes for high‐value species, but novel species with lower market values (i.e., many newly aquacultured species) or conservation programmes may struggle to fund the necessary large‐scale evaluations necessary for advanced breeding techniques; the same has proved true for genomic selection. Acknowledging this inequity, key next steps should include the development of a framework to evaluate the success of GO‐informed breeding decisions and simulating different strategies for integrating GO into breeding workflows, enabling comparative assessments of their performance. Such simulation efforts would help to ground‐truth the utility of GO and identify optimal strategies and workflows for initial empirical trials. For example, the possible use‐cases for GO in the context of OCS algorithms or as an independent trait in weighted selection indices could offer valuable feedback on the effectiveness and refinement of these approaches. By evaluating these potential applications through simulations, we can better understand the strengths and weaknesses of each approach to inform decisions about their implementation.

A key assumption underlying GO methods is that populations used to parametrise models are optimally adapted to current local environmental conditions (Gain et al. 2023). However, this assumption does not always hold true as populations may be disturbed or in a state of ‘adaptational lag’ to contemporary conditions (e.g., Browne et al. 2019). In a breeding programme context, populations that have been selectively bred for multiple generations or in cases where landscapes have been significantly altered by human activity, particularly for long‐lived species, disrupt the baseline for environmental associations and potentially lead to suboptimal breeding decisions. Future work should investigate how the underlying assumptions of various GO estimation methods might influence breeding applications and explore ways to address this limitation. Establishing best practices for parameterising models should be a priority for the future. Incorporating correction factors or historical data may prove useful for improving model performance, and simulation studies could be instrumental in resolving these challenges. Furthermore, future work should also consider what potential unintentional consequences that selecting for reduced GO values may incur in breeding populations. For example, there may exist trade‐offs for fitness or production traits in contemporary environmental conditions (Lee et al. 2024; Lundgren and Des Marais 2020; Willi and Van Buskirk 2022).

To overcome current limitations and enhance the utility of GO in selective‐breeding programmes, future research efforts should prioritise the development of comprehensive and standardised computational frameworks that integrate GO into current breeding practices. This will likely involve developing bioinformatic and analytical tools tailored specifically to breeding programme datasets and workflows and updating existing software to incorporate GO functionality (i.e., OCS algorithms). Experimentally evaluating these frameworks in model species, particularly in those with short generation times (e.g., chickens or tilapia), will provide empirical evidence to support the use of GO in breeding decisions. Because GO estimates are relative, the extent and diversity of sampling can influence their relevance. While comprehensive sampling of wild or domesticated populations would improve accuracy, this is often not feasible. Instead, priorities should be on maximising genetic and geographic diversity. For breeding programmes, this would encompass including all founding individuals as an estimate of within‐population variation. However, evaluation of how training data size and composition affect robust individual or population‐level GO estimates will be important for their application in selective breeding and conservation programmes.

Another intriguing future direction will be to explore the potential for an ‘epigenomic offset’ (Layton and Bradbury 2022). Epigenetic modifications, such as DNA methylation and chromatin structure changes, can significantly and much more rapidly influence gene regulation and trait expression within and among generations (Jaenisch and Bird 2003; Watson et al. 2021). Studies have shown that this epigenetic environmental memory can be inherited in diverse taxa across the tree of life (Anastasiadi et al. 2021). Incorporating epigenetic variation into breeding programmes represents an exciting avenue for future research (Gavery and Roberts 2017; Varotto et al. 2022). While promising, our current understanding of how epigenetic variation arises and is maintained, its evolutionary significance, and its ability to be inherited is still limited (Anastasiadi et al. 2021; Ashe et al. 2021; Silliman et al. 2023). More research is needed to explore how integrating epigenetic data could be applied in breeding programmes. If feasible, GO frameworks may present a useful tool for incorporating epigenetic variation and responses to environmental changes into breeding strategies.

## 5 Conclusions

Integrating GO into selective‐breeding programmes to create an *Adaptive Breeding Framework* offers a promising advancement at the intersection of evolutionary biology, landscape genomics and practical applications in agriculture and conservation. GO methodologies can improve the ability of plant and animal populations to respond to future environmental changes, moving beyond traditional strategies that focus primarily on current conditions. By addressing GxE interactions, GO can contribute to long‐term resilience and productivity, improving breeding efficiency and global food security.

Our exploration highlights several potential benefits of GO, including improved risk assessment, more informed breeding decisions for future environmental conditions, and strategic cryopreservation. GO can quantify the genetic changes needed for populations to maintain fitness in changing environments, offering a valuable metric for guiding selection and conservation efforts. Incorporating GO into established OCS algorithms or genomic selection indices may be a practical next step toward implementing this concept into existing breeding frameworks.

Before widespread adoption of GO, several challenges need to be addressed. GO methodologies are still evolving, with various statistical methods being validated in conservation settings. It will be important to refine these methods for breeding applications and develop standardised frameworks tailored to breeding needs. Importantly, the assumptions underlying GO models must be carefully evaluated to avoid suboptimal breeding decisions. Future research should focus on refining statistical frameworks for GO estimates and uncertainties, simulating integration strategies for GO with breeding programme operations, and empirically validating GO predictions in model species.

This article represents a thought‐experiment for how GO might be leveraged in agricultural settings. The application of this evolutionary tool offers a forward‐thinking approach to breeding programme management, ensuring genetic selection strategies are equipped to meet the demands of a rapidly changing environment. As methodologies are further developed and refined, GO has the potential to become a cornerstone of sustainable and resilient breeding programs. We hope that others will recognise these promising benefits and that this article will encourage the future funding and logistical support needed to bring these concepts into practice. Interdisciplinary collaboration among quantitative geneticists, evolutionary ecologists, breeders and bioinformaticians will foster innovative solutions for integrating GO into breeding programmes.

## Disclosure

Benefits generated: An international research collaboration was developed, including researchers from the USA, UK, Canada and New Zealand. The research herein addresses a priority concern for global food security, and our group is committed to fostering international scientific collaboration on this topic. All collaborators are included as coauthors.

## Conflicts of Interest

The authors declare no conflicts of interest.

## Data Availability

Data sharing not applicable to this article as no datasets were generated or analysed during the current study.
